# Immunomodulatory Effects of Chitotriosidase Enzyme

**DOI:** 10.1155/2016/2682680

**Published:** 2016-01-03

**Authors:** Mohamed A. Elmonem, Lambertus P. van den Heuvel, Elena N. Levtchenko

**Affiliations:** ^1^Department of Pediatric Nephrology & Growth and Regeneration, University Hospitals Leuven, KU Leuven, UZ Herestraat 49, P.O. Box 817, 3000 Leuven, Belgium; ^2^Department of Clinical and Chemical Pathology, Inherited Metabolic Disease Laboratory, Center of Social and Preventive Medicine, Faculty of Medicine, Cairo University, 2 Ali Pasha Ibrahim Street, Room 409, Monira, P.O. Box 11628, Cairo, Egypt; ^3^Department of Pediatric Nephrology, Radboud University Medical Center, Post 804, Postbus 9101, 6500 HB Nijmegen, Netherlands

## Abstract

Chitotriosidase enzyme (EC: 3.2.1.14) is the major active chitinase in the human body. It is produced mainly by activated macrophages, in which its expression is regulated by multiple intrinsic and extrinsic signals. Chitotriosidase was confirmed as essential element in the innate immunity against chitin containing organisms such as fungi and protozoa; however, its immunomodulatory effects extend far beyond innate immunity. In the current review, we will try to explore the expanding spectrum of immunological roles played by chitotriosidase enzyme in human health and disease and will discuss its up-to-date clinical value.

## 1. Introduction

Chitotriosidase enzyme (CHIT1, EC: 3.2.1.14), belonging to the family of 18 glycosyl hydrolases, was the first active chitinase to be discovered in human plasma [1]. Its natural substrate chitin is the second most abundant polysaccharide in nature after cellulose. Chitin is the linear polymer of N-acetylglucosamine and the main component of the cell walls of fungi and protozoa, egg shells of helminthes, and the exoskeletons of arthropods and insects; however, it is completely absent in mammals [2]. Several protein members of the same family were later detected in human plasma and tissues including the enzymatically active acidic mammalian chitinase (AMCase) [3] and chi-lectins, those having a chitin binding domain with no catalytic activity such as chitinase-3 like-1 protein (CHI3L1 or YKL-40), chitinase-3 like-2 protein (CHI3L2 or YKL-39), and oviductal glycoprotein-1 (OVGP1) [4].

In man, chitotriosidase is mainly expressed by different lineages of activated blood and tissue macrophages [5–10] and to a lesser extent by polymorphonuclear leucocytes [11]. The absence of its substrate chitin in the human body and the exclusive production by immunologically active cells immediately elicited the investigation of chitotriosidase involvement in the innate immunity against chitin coated pathogens [12]. Chitotriosidase was confirmed as an essential factor for the defense against many such organisms as* Plasmodium falciparum* [13],* Wuchereria bancrofti* [14],* Candida albicans* [15],* Madurella mycetomatis* [16], and* Cryptococcus neoformans* [17].

The role of chitotriosidase enzyme inside macrophages is not limited to its chitinolytic activity against the engulfed chitin containing organisms, or even to innate immunity. It has been implicated in the activation and polarization cascades of macrophages, as well as the indirect activation of other immune cells such as T helper cells and eosinophils [17–19]. Recent studies are interested in its immunomodulatory effects through the processes of chitin recognition, antigen presentation, induction of cell mediated immunity and synergistic effects with proteases, and other enzymes to kill different types of pathogens and cancer cells [10, 17–20]. On the other hand, chitotriosidase has been implicated in the pathogenesis of many human diseases through the improper induction of inflammation and faulty tissue remodeling such as bronchial asthma, chronic obstructive pulmonary disease (COPD), nonalcoholic fatty liver disease, and neurodegenerative disorders like Alzheimer's disease and amyotrophic lateral sclerosis [19, 21–24].

In the current review, we will provide a summary of basic information about the enzyme and we will discuss its immunomodulatory effects in humans over both innate and acquired immunity together with its current and possible future clinical applications.

## 2. Genetics

All the genes encoding active chitinases and chitinase-like proteins are clustered in two loci on the human chromosome 1 (1p13 and 1q32). This genetic clustering displays a high degree of conservation among different mammals indicating an evolutionary relationship through common ancestral gene duplication events [4].

**Figure 1:**
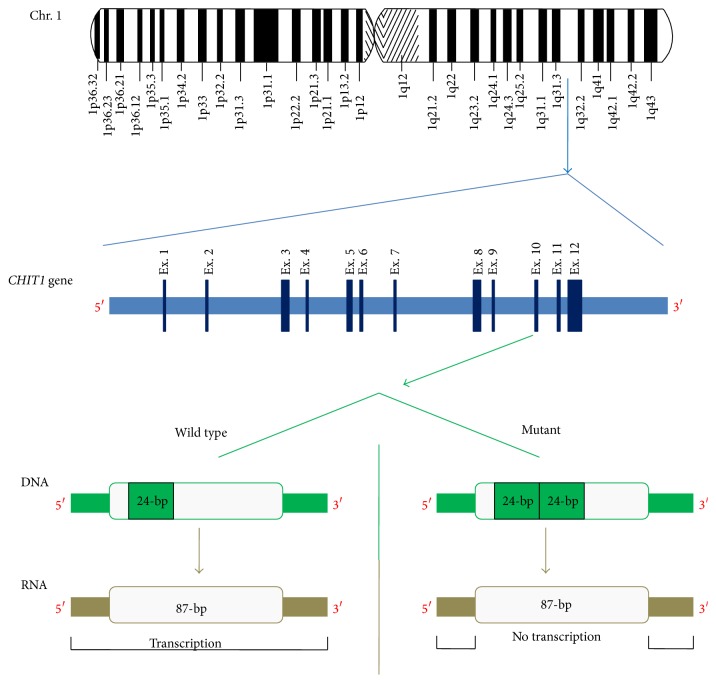
Schematic representation of human chromosome 1,* CHIT1* gene on chromosome 1q32 locus, and the common 24-bp duplication mutation at exon 10 of the* CHIT1* gene.

The human chitotriosidase gene* (CHIT1)*, extending over 20 kilobases at locus 1q32, consists of 12 exons translating a 466-amino acid protein [5, 25]. A common 24-bp duplication mutation in exon 10 of the* CHIT1* gene (Figure 1), leading to alternate splicing and in-frame deletion of 87 nucleotides (29 amino acids), is responsible for almost all detected enzyme deficiencies in different populations when homozygously mutated [25, 26]. Although the mutated 24-bp duplication allele is fairly common in Caucasian individuals (4–6% homozygous and 30–50% heterozygous), it is extremely rare in Sub-Saharan African individuals (0–2% only heterozygous), suggesting the evolutionary advantage of keeping the wild type enzyme in areas with high degrees of endemic parasitic and fungal disease loads [26]. Interestingly, the 24-bp duplication mutation is much more common in the Far East in populations of Japanese, Chinese, and Korean ancestries as almost 30% of them are homozygous, while 50% are heterozygous for the mutation [27, 28].

Other relatively common functional mutations have been also reported in the* CHIT1* gene like G102S, G354R, and A442V; however, when homozygously mutated, they are only associated with mild to moderate decrease in enzyme activity [27].

## 3. Chemistry and Modes of Action

Chitotriosidase enzyme has two major forms (a 50-kilodalton form dominant in blood stream and a 39-kilodalton form dominant in tissues), both having equal chitinolytic activities. The 50-kilodalton protein (466 amino acids) is initially produced by macrophages containing the 39-kilodalton N-terminus having the catalytic domain and the C-terminus having the chitin-binding domain connected together by a short hinge region. The 39-kilodalton protein (387 amino acids) is either cleaved post-translationally in the lysosome of macrophages or less commonly formed through differential RNA processing [25].

The enzyme was initially thought to be an exochitinase because it can hydrolyze chitotriose residues and was termed chitotriosidase based on this observation. However, recent structural and binding modes studies revealed the enzyme to be more of an endochitinase rather than an exochitinase [29, 30]. Furthermore, the strong binding affinity of chitotriosidase to its substrate is also responsible for the relatively high transglycosylation activity of the enzyme even in the absence of excess substrate concentrations, making chitotriosidase a complete independent chitinolytic machinery, and this is in accordance with its anticipated physiological role as a potent immunological defense weapon against microorganisms containing chitin [31, 32].

## 4. Stimulatory Signals

Although chitotriosidase enzyme is relatively recently linked to human pathology, many aspects of its intracellular and extracellular mechanistic actions, effector and affector molecules, and diseases influenced by the increase or decrease of its expression have been investigated. After its initial association with innate immunity against chitin coated pathogens it was rapidly identified as one of the major protein products of activated macrophages and hence an important nonspecific marker of macrophage activation [12]. Chitotriosidase activity was several hundred-fold elevated in the plasma of patients with the inflammatory based lysosomal storage disorder Gaucher's disease [1], in which macrophages play an essential role in the clearance of the disease sphingolipid storage material. The lipid laden macrophage becomes the disease histopathological pathognomonic cell in the bone marrow and tissues or what is known as the Gaucher cell. Furthermore, chitotriosidase is significantly elevated in some infections caused by bacterial and viral pathogens lacking its natural substrate chitin [33, 34].

**Figure 2:**
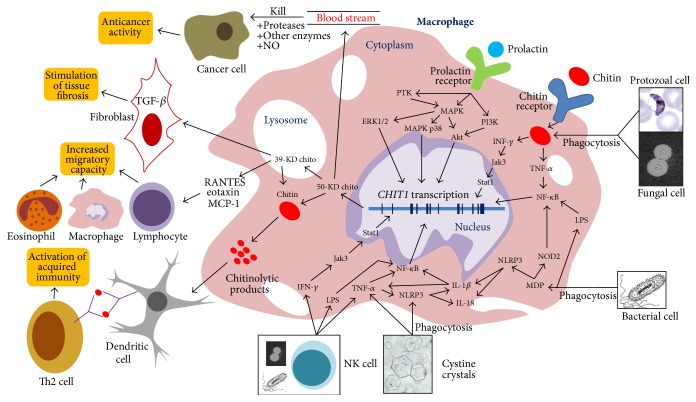
Schematic representation of a human macrophage showing different stimuli leading to the increased expression and release of chitotriosidase enzyme. An elaborate description of the processes implicated in increased chitotriosidase expression, as well as its immunological effects, is provided in the text. 39-KD chito: chitotriosidase (39 kilodalton protein); 50KD-chito: chitotriosidase (50 kilodalton protein); ERK1/2: extracellular signal regulated kinases 1/2; IL-1*β*: interleukin-1*β*; IL-18: interleukin-18; INF-*γ*: interferon-gamma; Jak: Janus kinase; LPS: lipopolysaccharide; MAPK: mitogen activated protein kinase; MCP-1: monocyte chemotactic protein-1; MDP: muramyl dipeptide; NF-*κ*B: nuclear factor-kB; NK cell: natural killer cell; NLRP3: NOD-like receptor family, pyrin domain containing 3; NO: nitric oxide; NOD2: nucleotide-binding oligomerization domain-containing protein 2; PI3K: PI3 kinase; PTK: protein tyrosine kinase; RANTES: regulated on activation, normal T cell expressed and secreted; Stat; signal transducers and activators of transcription; TGF-*β*: transforming growth factor-beta; Th2 cell: T helper type 2 cell; TNF-*α*: tumor necrosis factor-alpha.

Several intracellular pathways have been proposed to explain the stimulatory molecules and the activation cascades of chitotriosidase enzyme inside human macrophages.  Figure 2 provides a simple schematic representation of a macrophage with different proposed stimulatory signals for chitotriosidase expression as well as some of the main intra- and intercellular activities of the enzyme.

Chitin naturally is a potent activator of chitotriosidase expression, either phagocytized by the macrophage in the cell walls of different fungi and protozoa or directly introduced to macrophages through an unidentified receptor [35, 36]. Chitin and its small hydrolyzed particles when cocultured with macrophages can stimulate the production of TNF-*α* and IFN-*γ* [37] which can both increase the expression and activity of chitotriosidase enzyme inside macrophages [38]. Lipopolysaccharide (LPS) which is an important component of the bacterial cell wall was also confirmed as a potent stimulant of chitotriosidase transcription mainly through the NF-*κ*B signaling pathway [38, 39], which is also responsible for exerting the effects of TNF-*α*; however, IFN-*γ* most probably produces its effect through stimulating the Jak-Stat signaling pathway [40]. Another possible mechanism of chitotriosidase activation by bacteria is through the bacterial peptidoglycan product muramyl dipeptide (MDP), activating the NOD2 signaling pathway which is also implicated in the expression of chitotriosidase enzyme inside macrophages [41, 42].

Prolactin hormone which is structurally related to many human cytokines and is involved in the regulation of monocyte/macrophage functions was also shown to increase macrophage chitotriosidase production [43]. Through studying signal pathway inhibitors, prolactin was shown to stimulate chitotriosidase expression through multiple signaling pathways including the mitogen activated protein kinase (MAPK), PI3 kinase (PI3K/Akt), and the protein tyrosine kinase (PTK) pathways [44].

The inflammasome system is also expected to play some role in the activation of chitotriosidase expression as both ingested bacterial MDP and cystine crystals can stimulate macrophages through the NLRP3 inflammasome system [45, 46], leading eventually to the production of IL-1*β*, which either can stimulate the NF-*κ*B signaling pathway directly or indirectly induces the production of TNF-*α* [47], thus stimulating the expression of chitotriosidase.

Another efficient way to activate macrophages and induce chitotriosidase expression is through the paracrine effect of natural killer cells (NK cells), which when exposed to cells infected with viral, bacterial, or fungal pathogens or even neoplastic cells can produce large amounts of INF-*γ* and TNF-*α* [36, 48] in the vicinity of macrophages, both increasing the expression and release of chitotriosidase.

## 5. Immunological Effects and Clinical Perspectives

Chitotriosidase enzyme expression increases exponentially during the normal monocyte to macrophage maturation process showing a peak of expression between the 5th day and the 7th day of culture [18] and is recently detected to be expressed in both macrophage polarization forms (M1 and M2). M1 macrophages, or classically activated macrophages, are mainly directed to promote inflammation, kill the invading pathogens. and stimulate tissue fibrosis following injury. On the other hand, M2 macrophages, or alternatively activated macrophages, provide regulatory signals to protect the host from an exaggerated inflammatory response and promote tissue remodeling and healing [49]. The fact that chitotriosidase is expressed almost equally in both forms denotes its regulatory roles over processes far beyond the hydrolysis of chitin in pathogens. Further studies are still needed to clarify the role of chitotriosidase enzyme in the alternatively activated M2 macrophages.

Dendritic cells are the most important antigen presenting cells in the human body and one of the members of the monocyte/macrophage lineage. Although AMCase is widely expressed in different tissues, especially in the epithelial cells of the gastrointestinal tract and lungs [3], chitotriosidase was the only implicated active chitinase in the process of chitin recognition and antigen presentation [17]. Recent evidence suggests that the induction of human T helper 2 cells (Th2) in response to pulmonary cryptococcal infection is totally dependent on chitin cleavage by chitotriosidase and that CD11b+ conventional dendritic cells act as antigen presenting cells for the specifically fragmented chitin products [17]. Furthermore, chitotriosidase mRNA and protein concentrations were significantly elevated in mature dendritic cells without chitin sensitization as compared to immature dendritic cells [10], implying that chitotriosidase might be also playing a role in the process of antigen presentation regardless of the presence of chitin.

Recently, activated macrophages and chitotriosidase elevations were implicated in the pathogenesis of nephropathic cystinosis, another lysosomal storage disorder characterized by cystine crystal accumulation inside macrophages in different body organs. Cystine crystals* in vitro* when incubated with human monocyte derived macrophages were able to activate macrophages in a concentration dependent manner evidenced by the increased concentrations of TNF-*α* and the concomitant activities of chitotriosidase enzyme in culture supernatant and in cell homogenate. Furthermore, plasma chitotriosidase activities in cystinotic patients correlated positively with leucocytes cystine concentrations, making it a potential target for the disease therapeutic monitoring [50]. Cystinosis was the first crystal based disease with the confirmed involvement of chitotriosidase enzyme in its pathogenesis, making it an interesting target to investigate in other more common crystal related disorders such as gout and hyperoxaluria.

Chitotriosidase also mediates many inflammatory processes through the direct stimulation of different inflammatory mediators such as IL-8, MMP9 (collagenase type IV), MCP-1 (CCL2), RANTES (CCL5), and eotaxin (CCL11), thus increasing the migratory capacity of many immunological cells including T lymphocytes, macrophages, and eosinophils [51, 52]. Levels of chitotriosidase activities strongly correlated with the concentrations of IL-1*β* and TNF-*α* in the bronchoalveolar lavage (BAL) of COPD patients supporting the hypothesis of a mutual regulation cascade in the production of these inflammatory mediators [52]. Furthermore, chitotriosidase was involved in the induction of fibrosis in the murine model of interstitial lung disease as the bleomycin-induced pulmonary fibrosis was significantly reduced in *Chit*1^−/−^ mice and significantly enhanced in lungs from* Chit1* overexpressing transgenic mice. This effect is explained by the activation of fibroblasts through enhancing TGF-*β* and increasing the expression of TGF-*β* receptors 1 and 2 leading to the activation of the Smad and MAPK/ERK signaling pathways [53]. Similar effects have been clinically observed with other human diseases characterized by faulty tissue remodeling and abnormal healing such as nonalcoholic fatty liver disease, bronchial asthma, and diabetic nephropathy in which chitotriosidase plasma activities strongly correlated with disease progression and/or the degree of tissue fibrosis [21, 22, 54]. Targeting the chitotriosidase activation cascade through the administration of specific antibodies or pan-chitinase inhibitors significantly ameliorates inflammation and fibrosis in several animal models of autoimmune diseases, most likely by suppressing the chitotriosidase dependent release of different cytokines and chemokines [55]; however, the safety of this approach in humans is not yet determined as it might increase the susceptibility to fungal and protozoal infections.

Chitinases and chi-lectins could play a detrimental role in human cancer development, especially CHI3L1 (YKL-40) which has been associated with increased tumor angiogenesis and bad prognosis in many human neoplasms such as breast, lung, and cervical cancers [56–58]. On the other hand, chitinases are also believed to have some anticancer cell activities [59]. Macrophages were always considered as a primary defense line against neoplastic cells, but the exact mechanisms beyond this action were not very clear [60]. Speculations were made about a combined effect of released NO and H_2_O_2_; however, there was no much evidence to support this hypothesis [20]. Recently, bacterial and human chitinases were both confirmed as having strong synergistic effects with protease enzymes produced by macrophages to dissolve mucin [61]. This mucolytic activity selectively attacked the altered mucin in the cancer cell wall of animal models and not the healthy cell mucin [59]. It is too early to speculate about the therapeutic applications of this observation as many explanatory mechanistic studies are needed to determine the specificity and the exact molecular and chemical targets of this process.

Chitotriosidase is currently an established or a candidate screening marker, severity marker, and/or therapeutic monitor for over 40 different diseases, inherited and acquired.  Table 1 provides a summary of human diseases clinically associated with macrophage activation and chitotriosidase enzyme production. Being a nonspecific marker of macrophage activation and deficient in about 6% of normal population clearly limits chitotriosidase usability as a screening marker for many diseases; however, in established diagnosed nondeficient patients, chitotriosidase is an excellent marker to monitor compliance and response to treatment [62, 63], especially when the treatment targets inflammatory pathways. And in some diseases its marked elevations made it also a quite beneficial screening marker as in Gaucher's disease even when other lysosomal storage disorders are suspected [64].

## 6. Conclusions

Being a sensitive biomarker of macrophage activation, chitotriosidase activity is currently more and more commonly used in clinical practice to evaluate the status and response to treatment of inflammatory based diseases in which macrophages play a significant role. The interesting mixture of harmful and beneficial immunological effects made human chitinases and chitotriosidase enzyme an intriguing point of research. If proven safe in humans, the development of targeted therapies either to suppress chitotriosidase activity in autoimmune and inflammatory disorders, or to specifically enhance its targeted activity to kill cancer cells or to potentiate immunity against certain infections will not be far in the future.

## Figures and Tables

**Table 1:** Human diseases associated with elevated chitotriosidase enzyme.

Disease group	Disease	Proposed clinical value (sample type)	References
Lysosomal storage diseases	Gaucher	Screening, therapeutic monitoring (P/S)	[1, 62]
	Niemann-Pick A/B and C	Screening, therapeutic monitoring (P/S)	[65]
	Cystinosis	Therapeutic monitoring (P/S)	[50]
	Fabry	Therapeutic monitoring (P/S)	[66]
	Krabbe	Screening, marker of severity (P/S)	[67]
	Wolman	Therapeutic monitoring (P/S)	[68]
	Farber	Screening (P/S)	[69]
	GM1	Screening (P/S)	[70]
	Sialidosis type II	Screening (P/S)	[71]

Infectious diseases	Systemic fungal infections: *Candida albicans, Madurella mycetomatis, and Cryptococcus neoformans*	Prognosis, therapeutic monitoring (P/S)	[15–17]
	Malaria	Prognosis (P/S)	[13]
	Filariasis	Screening (P/S)	[14]
	Tuberculosis	Prognosis, therapeutic monitoring (P/S)	[72]
	Brucellosis	Therapeutic monitoring (P/S)	[73]
	Leprosy	Prognosis, therapeutic monitoring (P/S)	[74]
	Crimean-Congo hemorrhagic fever	Prognosis (P/S)	[34]

Respiratory diseases	Asthma	Marker of severity (P/S)	[19, 21]
	COPD	Marker of severity (P/S, BAL)	[21, 51]
	Interstitial lung disease	Screening, marker of severity (BAL)	[52, 75]

Endocrinological diseases	Diabetes	Marker of endothelial damage (P/S)	[76]
		Marker of nephropathy progression (P/S)	[53]

Cardiovascular diseases	Atherosclerosis	Marker of severity (P/S)	[77, 78]
	Stroke	Prognosis (P/S)	[79]
	Coronary artery disease	Prognosis (P/S)	[80]
	Erectile dysfunction	Marker of severity (P/S)	[81]

Neurological diseases	Amyotrophic lateral sclerosis	Screening, marker of severity (P/S, CSF)	[24, 82]
	Alzheimer's disease	Prognosis, marker of severity (CSF)	[23, 83]
	Cerebral adrenoleukodystrophy	Prognosis (P/S, CSF)	[84]
	Neuromyelitis optica	Screening (CSF)	[50]
	Multiple sclerosis	Screening, prognosis (CSF)	[50]

Gynecological and obstetrical diseases	PCOS	Prognosis (P/S)	[85]
	Endometriosis	Marker of severity (P/S)	[86]
	Preeclampsia	Marker of fetal compromise (UC)	[87]

Miscellaneous	NAFLD	Marker of severity (P/S)	[22]
	FMF	Screening, marker of severity (P/S)	[88]
	*β*-Thalassemia	Marker of severity, therapeutic monitoring (P/S)	[89]
	Sarcoidosis	Marker of severity, therapeutic monitoring (P/S)	[63]
	Acute appendicitis	Screening (P/S)	[90]
	Juvenile idiopathic arthritis	Screening, marker of severity (SV)	[91]
	Prostate cancer	Prognosis (P/S)	[92]

BAL: bronchoalveolar lavage; COPD: chronic obstructive pulmonary disease; CSF: cerebrospinal fluid; FMF: familial Mediterranean fever; GM1: gangliosidosis M1; NAFLD: nonalcoholic fatty liver disease; PCOS: polycystic ovarian syndrome; P/S: plasma or serum; SV: synovial fluid; UC: umbilical cord blood.
